# The Brain Ages Optimally to Model Its Environment: Evidence from Sensory Learning over the Adult Lifespan

**DOI:** 10.1371/journal.pcbi.1003422

**Published:** 2014-01-23

**Authors:** Rosalyn J. Moran, Mkael Symmonds, Raymond J. Dolan, Karl J. Friston

**Affiliations:** 1 Virginia Tech Carilion Research Institute and Bradley Department of Electrical & Computer Engineering, Roanoke, Virginia, United States of America; 2 Wellcome Trust Centre for Neuroimaging, Institute of Neurology, University College London, London, United Kingdom; 3 Nuffield Department of Clinical Neurosciences, Oxford University, John Radcliffe Hospital, Oxford, United Kingdom; Indiana University, United States of America

## Abstract

The aging brain shows a progressive loss of neuropil, which is accompanied by subtle changes in neuronal plasticity, sensory learning and memory. Neurophysiologically, aging attenuates evoked responses—including the mismatch negativity (MMN). This is accompanied by a shift in cortical responsivity from sensory (posterior) regions to executive (anterior) regions, which has been interpreted as a compensatory response for cognitive decline. Theoretical neurobiology offers a simpler explanation for all of these effects—from a Bayesian perspective, as the brain is progressively optimized to model its world, its complexity will decrease. A corollary of this complexity reduction is an attenuation of Bayesian updating or sensory learning. Here we confirmed this hypothesis using magnetoencephalographic recordings of the mismatch negativity elicited in a large cohort of human subjects, in their third to ninth decade. Employing dynamic causal modeling to assay the synaptic mechanisms underlying these non-invasive recordings, we found a selective age-related attenuation of synaptic connectivity changes that underpin rapid sensory learning. In contrast, baseline synaptic connectivity strengths were consistently strong over the decades. Our findings suggest that the lifetime accrual of sensory experience optimizes functional brain architectures to enable efficient and generalizable predictions of the world.

## Author Summary

While studies of aging are widely framed in terms of their demarcation of degenerative processes, the brain provides a unique opportunity to uncover the adaptive effects of getting older. Though intuitively reasonable, that life-experience and wisdom should reside somewhere in human cortex, these features have eluded neuroscientific explanation. The present study utilizes a “Bayesian Brain” framework to motivate an analysis of cortical circuit processing. From a Bayesian perspective, the brain represents a model of its environment and offers predictions about the world, while responding, through changing synaptic strengths to novel interactions and experiences. We hypothesized that these predictive and updating processes are modified as we age, representing an optimization of neuronal architecture. Using novel sensory stimuli we demonstrate that synaptic connections of older brains resist trial by trial learning to provide a robust model of their sensory environment. These older brains are capable of processing a wider range of sensory inputs – representing experienced generalists. We thus explain how, contrary to a singularly degenerative point-of-view, aging neurobiological effects may be understood, in sanguine terms, as adaptive and useful.

## Introduction

Aging is generally thought to be accompanied by reduced neuronal plasticity and a loss of neuronal processes that accounts for a loss of grey matter, which progresses gently with age [1]–[3]. Many concomitants of physiological aging have been studied. In particular, studies of the mismatch negativity (MMN) speak to a decline in sensory learning or memory [4], [5]. For example, elderly subjects show a significant reduction in superior temporal gyrus responses, which has been interpreted as “an aging-related decline in auditory sensory memory and automatic change detection” [6]. In this work, we examine the physiological basis of attenuated mismatch responses using dynamic causal modeling in a large cohort of human subjects. However, we motivate the present study using an alternative – and slightly more optimistic – model of normal aging.

**Figure 1:**
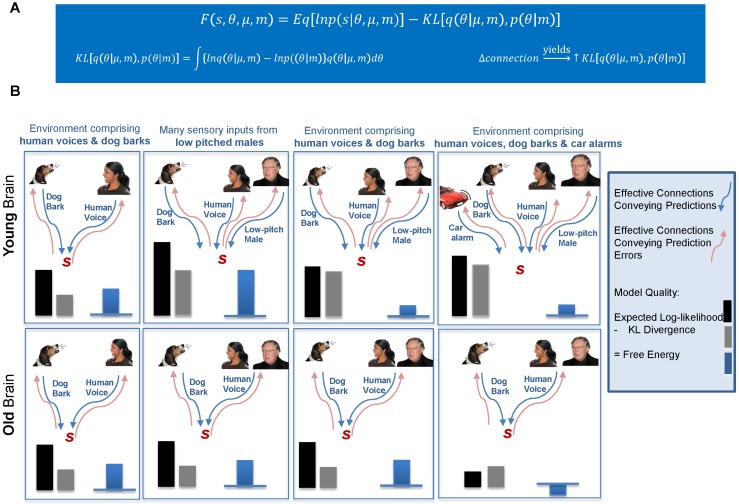
Hypotheses – explanations for sensory input. A) The Negative Free Energy (*F*) is maximized by the brain (model, *m*) to ensure homeo/allostasis. An optimal model can accurately predict incoming sensory signals *s*, (this accuracy term is the expected log-likelihood of the sensory signal *s*, under the conditional density, *q* ie. 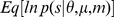 ) while ensuring generalization, when inferring new sensory causes (θ represented through their sufficient statistics *μ*). This complexity penalty 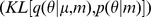 is revealed during the presentation of the oddball. Given changes in synaptic efficacy of forward connections; i.e. learning the standard tone - the Kullback-Leibler (KL) divergence between the learned prior, *p* and the posterior, *q* under these new (oddball) data will be high. These effects, indicating brittle models, were hypothesized to be less pronounced in older subjects. B) An illustration of how model optimality depends on the environment. Left-most panels: In a constant environment both young (top) and old (bottom) brains have connections that convey accurate predictions (blue arrows). The sensory input, s, will result in prediction error messages (red arrows) that are cancelled by the appropriate prediction. A change in the environment (e.g., from a dog bark to a human voice) will result in prediction error signals along the human voice pathway until human voice predictions are made and cancellation occurs. This type of predictive coding scheme has been proposed as the mechanism underlying the mismatch negativity [17]. In this scenario, both young and old brains generate accurate predictions with similar complexity. Centre Left panels: repeated sensory input from a specific human voice results in new prediction and error pathways for that particular vocalization in a younger brain. For this environment, the younger brain is more accurate (at the penalty of higher complexity) and may outperform the older brain in terms of model quality. Centre Right panels: on return to the original environment, the older brain – that has maintained a less complex model – outperforms the younger brain. Right-most panels: In a novel environment that persists, younger brains – that support more flexible Bayesian updating – will outperform older brains. In this context, the degrees of freedom subtended by effective connections in the older brain are not sufficient to simulate the environment and provide accurate predictions.

Our basic premise is that aging reflects a progressive refinement and optimization of generative models used by the brain to predict states of the world – and to facilitate an active exchange with it. Evidence that the brain learns to predict its environment has been demonstrated in the perceptual [7], motor [8] and cognitive domains [9]. These studies are motivated by formal theories – such as the free energy principle and predictive coding – that appeal to the Bayesian brain hypothesis [10]–[14]. In this theoretical framework [12], the quality of the brain's model is measured by Bayesian model evidence. Crucially, model evidence can be expressed as accuracy minus complexity. This means that as the brain gets older – and maintains an accurate prediction of the sensorium – it can progressively improve its performance by decreasing its complexity. This provides a normative account for the loss of synaptic connections and fits intuitively with the notion that as we get older we get wiser, more sanguine and ‘stuck in our ways’. Formally, under the Free Energy Principle, the brain supports active exchanges with the environment in order to minimize the surprise associated with sensory inputs. Over time, learning optimizes brain connectivity to support better predictions of the environment [15]. These ‘better’ models must conform to Occam's razor by providing accurate predictions with minimal complexity [16]. In Figure 1 we illustrate model qualities prescribed by the Free Energy Principle, potential age effects and their context or environmental sensitivity. This formulation of Free Energy minimization is based on hierarchical message passing and predictive coding. Neuronal implementations of predictive coding have been proposed as the mechanisms underlying the MMN [17], [18]. In the present study, we address the corollary of model complexity minimization; namely, less reliance on Bayesian updating through sensory learning and underlying neuronal plasticity. Mathematically, an attenuation of Bayesian learning precludes overfitting of sensory data; thereby minimizing complexity and ensuring that explanations for those data generalize. In other words, as we age, we converge on an accurate and parsimonious model of our particular world (Figure 1B) - whose constancy we actively strive to maintain (Figure 1B). Its neuronal implementation would be consistent with a large literature on synaptic mechanisms in aging and a progressive decline in neuromodulatory (e.g., dopaminergic [19], [20]) activity that underwrites changes in synaptic efficacy [21].

The implications for the neurobiology of aging are that – over the years – cortical message passing may become more efficient (providing accurate predictions with a less redundant or complex hierarchical model) and increasingly dominated by top-down predictions. This is consistent with reports of age-induced shifts in neuronal activation from sensory to prefrontal regions [22]. The hypothesis addressed in the present study was that the Bayesian updating implicit in the sensory learning of standard stimuli in the MMN paradigm would fall progressively with age. In particular, we predicted that changes in effective connectivity during the processing of repeated stimuli (namely, changes in forward connections to superior temporal cortex) would be attenuated as a function of age.

Here, we examined age-related attenuation of sensory learning by quantifying synaptic coupling or effective connectivity changes using the mismatch negativity (MMN) paradigm and dynamic causal modeling (DCM). There is a large literature on DCM and the MMN [23]–[25], where changes in coupling during repetition of standard stimuli are revealed by differential responses to oddball stimuli – producing the MMN (oddball minus standard) difference in event related potentials that peaks around 150 msec. These connectivity changes (plasticity) are expressed in both intrinsic connections within auditory sources and in an increase in the effective connectivity from auditory to superior temporal sources during the processing of oddball relative to (learned) standard stimuli [26]. These changes have been interpreted in terms of predictive coding, in which bottom-up or ascending prediction errors (under modulatory gain control) adjust representations at higher levels in the cortical hierarchy – that then reciprocate descending predictions to cancel prediction error at lower levels.

Recent studies of age-related changes in functional connectivity provide evidence for changes in long-range coupling with age [27]. Our hypothesis rests on changes in (directed) effective connectivity that produces the functional connectivity or dependencies in measured activity [28]. To quantify changes in effective connectivity we used DCM [29] to model magnetoencephalographic (MEG) recordings. DCM uses forward models of evoked responses based on neuronal mass formulations that account for the laminar specificity of forward and backward connections [29]. These models have been previously validated using animal [30] and human recordings [31], and provide subject-specific measures of intrinsic (within source) and extrinsic (between source) synaptic coupling.

## Results

### Dynamic Causal Modeling of Sensory Evoked Responses

**Figure 2:**
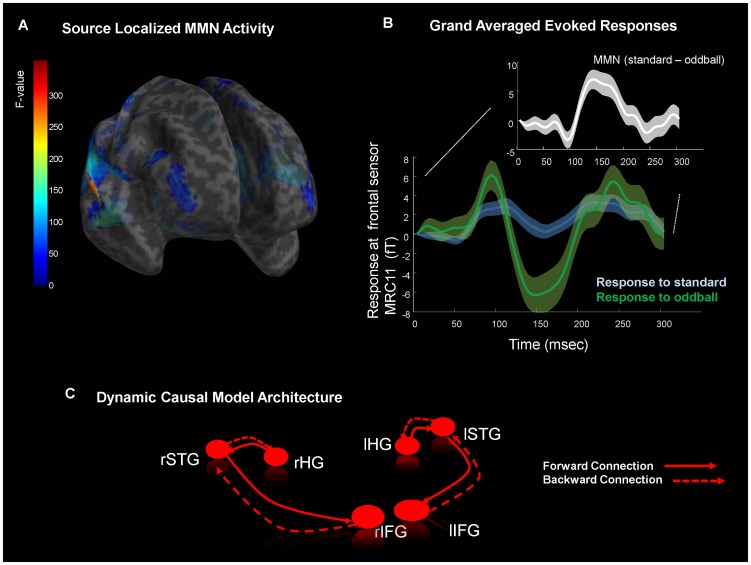
Mismatch Network. A) Statistical parametric mapping of mismatch (standard – oddball) effect across subjects (*p*<0.05 *FWE* corrected) sharing a color-coded *F* statistic on a semi-transparent canonical cortical inflated mesh. This SPM compares the power (in frequencies from 0–30 Hz, over 60–300 msec of peristimulus time), evoked by oddball stimuli with the equivalent power evoked by standard stimuli. B) Auditory evoked responses recorded at one MEG sensor over right frontal cortex. Plotted are the grand averaged evoked measurements across all sessions (shaded areas represent their standard deviation) in response to standard tones (blue) and oddball tones (green). The difference in these responses constitutes the mismatch negativity (MMN); seen here as the negative differences from 100–200 msec (white inset) – as predicted from the literature. Both types of trials were fitted for each subject in the DCM analysis. C) In the DCM, we modeled the transmission of neuronal activity from primary sensory to frontal regions using three sources reciprocally connected in each hemisphere; source location priors were as follows: left HG: x = −42, y = −22, z = 7; right HG: x = 46, y = −14, z = 8; left STG: x = −61, y = −32, z = 8; right STG: x = 59, y = −25, z = 8; left IFG: x = −46, y = 20, z = 8; right IFG: x = 46, y = 20, z = 8. Inputs entered Heschl's gyrus bilaterally and were passed via forward connections to STG within each hemisphere. STG sent top-down backward connections to HG. STG also sent forward connections up to IFG and received backward connections from IFG. Each source is modeled in the DCM with a neural mass model. The parameters of synaptic interactions within each source, as well as the extrinsic connections between sources were optimized during model inversion. The extrinsic connectivity was equipped with an additional parameter that allowed for different connection strengths during standard or oddball stimulus processing.

We measured event-related MEG responses in 97 subjects, aged 20 to 83 and applied DCM to quantify the underlying synaptic coupling producing observed responses. We used an auditory oddball paradigm to elicit the mismatch negativity or MMN [23]. Our stimuli comprised pseudo-random tone sequences, with standard (frequent) tones interspersed with infrequent oddball tones (with a presentation frequency of 88% and 12% respectively). Consistent with previous studies of MMN generation [32], [33], source localization revealed hierarchical responses (Figure 2A), with large magnitude responses in auditory, temporal and inferior frontal sources (*p<0.05* family-wise error corrected; Figure 2A). A prominent MMN (oddball – minus standard) was observed, as expected, around 150 msec post stimulus (Figure 2B).

Following previous DCM studies of the MMN, we used a six–source model to characterize age effects within the MMN network (Figure 2C). For each subject, we inverted the ensuing DCM to obtain subject-specific measures of (changes in) connectivity based on their evoked responses to standards and oddballs. In this DCM, auditory input enters bilaterally at Heschl's gyrus (HG), these primary auditory sources were connected via forward connections to superior temporal gyrus (STG) sources, which in turn sent forward connections to the inferior frontal gyrus (IFG). Reciprocal backward connections were included to allow signal propagation down the hierarchy from IFG to STG and from STG to HG (Figure 2C). Each source was modeled with a neural mass model comprising three neuronal populations, with distinct receptor types and intrinsic connectivity [31]. Specifically, the model contains synaptic parameters that encode the contribution of AMPA, NMDA and GABAa receptor mediated currents in three populations: comprising pyramidal cells, inhibitory interneurons and granular-layer spiny-stellate cells. These populations are connected intrinsically and receive extrinsic inputs according to their laminar disposition: forward connections drive spiny stellate cells and backward connections drive pyramidal cells and inhibitory interneurons [29]. Crucially, we included stimulus-specific parameters that changed the strength of extrinsic connections when responding to standard and oddball inputs. This enabled us to test our hypothesis of age-related differences in connectivity changes. Specifically, we hypothesized that the learning or repetition-dependent increase in sensitivity to extrinsic forward afferents – conveying prediction errors induced by the oddball events – would be attenuated in older subjects.

**Figure 3:**
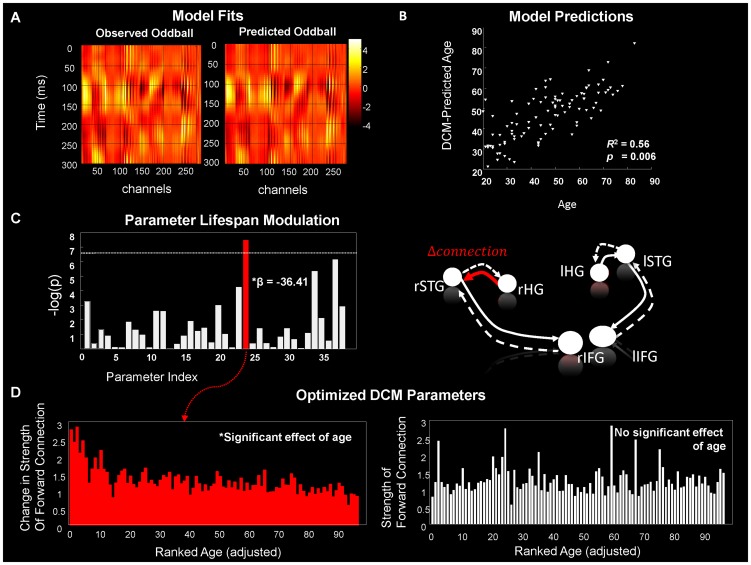
Age Effects from DCM's Neuronal Parameters. A) Representative example of data fit shown as a sensor space image for all MEG channels (along the x-axis) over peristimulus time (0–300 msec along the y-axis). Data are normalized to arbitrary units according to color bar. B) Subjects age as predicted by a linear regression on the DCM neuronal parameters. C) Left: Contribution of each parameter to the regression: negative log *p*-value for all 38 regression coefficients (37 DCM parameters and a constant; Table 1) as assessed using the appropriate *t*-statistic. The horizontal line depicts the Bonferroni-corrected significance level. One parameter has a significant *p*-value: this parameter encoded the difference in forward connectivity to right STG, between oddballs and standard and had a negative correlation with age. Right: Red is the forward connectivity parameter, illustrated within the DCM architecture, where age was predicted. D) Individual DCM parameter estimates. Left: the parameter controlling changes in connectivity from right HG to right STG identified above, plotted according to an adjusted (for the effect of remaining parameters in the regression model} age ranking. Right: a similar plot illustrating the latent connectivity strength from right HG to right STG, plotted according to age rank, adjusted as above.

An analysis of model fits confirmed that DCM provided an accurate account of the evoked responses (193 data sets were inverted in total), accounting for 81%±12% (mean ± std) of the empirical variance (for a representative example see Figure 3A). We found no evidence for age-dependent differences in model fit (*p*>0.1, Pearson correlation of age and proportion of variance explained).

### Neuronal Parameters Predicting Age

Having established the accuracy of the DCM, we then asked whether the subjects' age could be predicted by neuronal parameters that included: i) the strength of forward and backward extrinsic connections, ii) changes in these connections during oddball (compared to standard) tones, iii) the strength of intrinsic connections within each source, iv) parameters controlling synaptic adaptation; namely, time constants of AMPA, NMDA and GABAa receptors, membrane capacitance, subcortical input strength and axonal delays (37 parameters and a constant term see Table 1). Electromagnetic lead field parameters were optimized for each DCM but not included in this predictive analysis (see Methods).

**Table 1:** Neuronal parameters of the DCM – description and prior values presented in Figure 3C.

Parameter (Parameter Index in Figure 3C)	Physiological Interpretation	Prior:	
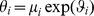		Mean: 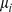	Variance: 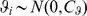
*S (2)*	*Parameter controlling covariance amongst states (optimized for all sources)*	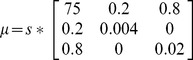	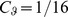
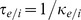 *(3–8)*	*Average synaptic time-constant AMPA-like channels (optimized per source)*	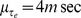	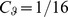
	*GABAa-like channels*	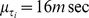	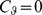
	*NMDA like channels*	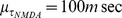	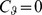
*G (9–14)*	*Intrinsic Excitatory Connectivity (optimized per source)*	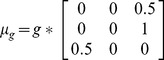	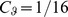
*A (15–18)*	*Extrinsic Forward Connection*	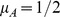	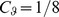
*A (19–22)*	*Extrinsic Backward Connections*	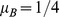	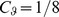
*B (23–30)*	*Modulations of Extrinsic Connection*	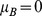	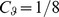
*C (31–32)*	*Input Strength of volley from Thalamus to Left and Right Primary Auditory Cortex*	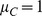	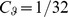
*R1 (33)*	*Controls the size of the input volley (a Gaussian bump function) from the thalamus, onset: 64 msec*	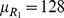	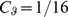
*R2 (34)*	*Controls the duration of the input volley*	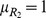	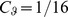
*d (35)*	*Intrinsic conduction delay*	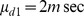	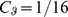
*(36)*	*Extrinsic conduction delay*	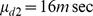	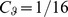
*U (37)*	*Background Synaptic Input*	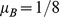	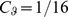
*CV (38)*	*Membrane Capacitance*	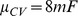	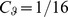

Note parameters are log-scaling parameters: 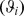. These operate on variables with the following prior mean – and can be found in spm_fx_nmda.m, part of the DCM_MEG toolbox in SPM (http://www.fil.ion.ucl.ac.uk/spm/).

(* indicates age-predictive parameter).

Using a multiple linear regression, we found that the neuronal DCM parameters could predict age with a high degree of reliability (*R^2^* = 0.56; F_37,59_ = 2.06; *p* = 0.006; Figure 3B). Post-hoc *t*-tests were used to identify the parameters with the greatest predictive ability. Across all regression coefficients, the largest and only significant regression coefficient (correcting for 38 tests) was associated with the learning dependent increase in forward connectivity from the right primary auditory cortex to the right superior temporal gyrus (β = −36.41; *p*<0.05 Bonferroni corrected, Figure 3C). This increase was attenuated over the lifespan, speaking to a reduced sensitivity of STG responses to ascending (prediction error) afferents from primary auditory cortex. This was in contradistinction to the latent connectivity strengths from right primary auditory to superior temporal gyrus - that do not reflect learning – which were consistent across the lifespan population (Figure 3D).

### Complexity Minimization under the Free Energy Principle

The Free Energy Principle [11] provides a description of neurobiological circuit processing that attributes specific computational roles to forward, backward, lateral (extrinsic) connections and intrinsic connections and their neuromodulation [34]. Each level of a processing hierarchy transmits predictions to the level below, which reciprocates with bottom-up prediction errors. Bayes optimal perception and action is achieved by maximising the Negative Free Energy (*F*):
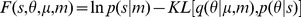
(1)Maximising this functional at every point in time ensures homeo/allostasis [12], by minimising the surprise (the negative log model evidence 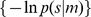) of incoming sensory signals *s* caused by states of the world 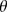, represented in the brain with their sufficient statistics *μ.* It renders the current prediction of states of the environment; 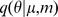, close to the true probability of those states; 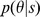 (where the distance measure is the Kullback-Leibler divergence *KL*). This process is dependent on the model the brain instantiates, *m*. Rearranging this equation, we see that the quality of this model can be decomposed into two components; representing accuracy and complexity.
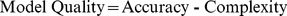

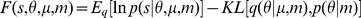
(2)In our connectivity analysis, the only consistent aging effect was manifest in trial-by-trial updates and revealed during the presentation of the oddball. This is represented mathematically as the KL-divergence from the approximate posterior to the prior, ie. the complexity penalty; which reduced over the lifespan (Figure 1). Over-learning of the standard tone by younger subjects is indicative of brittle models. These effects were significantly less pronounced as our cohort (cross-sectionally) aged. In contrast, accuracy was equivalent across the lifespan on a trial-average basis, since younger subjects learned the standard tone; indicating poor predictions to early standard and all deviant tones with better predictions to later standard tones; while age induced greater baseline predictions overall, that were generalizable to auditory deviants.

## Discussion

These results are interesting for two reasons. First, the ability of subject-specific DCM parameters to predict age in such a reliable way suggests that the coupling estimates have a high degree of predictive validity. Second, it is remarkable that the most predictive parameter encoded a sensory learning effect – as opposed to a connection engaged by the predictive coding of standard or oddball stimuli *per se*. Furthermore, the particular connection implicated – the forward primary auditory afferent to STG – has been found to increase in previous DCM studies of the MMN [26]. The present study is the largest DCM study reported to date and underscores a general point; namely, that biologically grounded models of evoked responses can disclose important associations between quantitative estimates of functional brain architectures and the behavioral or clinical phenotype. In particular, we used our data to estimate the underlying causes of evoked responses – and did not simply look for correlations between age and a particular data feature (e.g., the MMN magnitude). This means that we could account for a range of potentially age-related confounds (e.g., intersubject differences in lead fields) that would otherwise obscure structure-function relationships of interest.

In conclusion, our results suggest that effective connectivity in the human brain does not undergo indiscriminate age-related decline but shows a selective and specific attenuation of plasticity in the face of short-term sensory learning or memory. In other words, there were no systematic age-related changes in effective connectivity when processing auditory stimuli *per se*. This is consistent with the conjecture that older brains are more efficient (less complex) models of the sensorium and are less predisposed to short-term (Bayesian) updating.

The present study was motivated by recent perspectives provided by theoretical neurobiology [12] that offer a principled explanation for the reduction in connectivity (complexity) with progressive optimization of the generative models the brain uses for hierarchical Bayesian inference. A corollary of this complexity minimization is decreased Bayesian updating and neuroplasticity that we confirmed experimentally with a sensory learning (oddball) paradigm. Our results may call for a reinterpretation of aging neuroimaging studies; in particular, the *compensation hypothesis* that has been provided as explanation for age-related changes in the pattern of cortical activations [22], [35], [36]. Indeed, a reinterpretation has been offered from a cognitive perspective [37] where a shift from bottom-up to top-down processing has been proposed to explain better cognitive performance in older individuals [38]. These performance gains have been shown to accrue in unconventional (generalized) re-test circumstances; e.g. using distractors that should have been ignored in one task, to complete later tasks [39]. From the perspective of task performance, complexity reduction would similarly support reliability, as exhibited by older participants in a recent study of performance consistency across multiple cognitive domains [40]. The complexity minimization perspective may also account for de-differentiation in cortical specialization [41]–[43] and cognitive structure [44], [45] due to age - in the sense that simpler generative models require fewer degrees of freedom (functional specialization) to predict sensorimotor contingencies. While our results focus on functional connections, structural changes commensurate with complexity reduction have recently been demonstrated in a non-aged but practiced cohort of ballerinas. In their study [46], highly trained ballet dancers - who show improved stability in response to spinning - exhibited grey matter reductions in cerebellar grey matter compared to controls. Furthermore, controls showed enhanced vestibular perception that was positively correlated with cortical white-matter measures, an effect absent in the dancers, effects summarized by the authors as “training-related attenuation”.

Interestingly, the schema presented in Figure 1 was supported by learning effects in early sensory cortex. These were constrained to the right hemisphere, where classical MMN effects are most pronounced [47]. Complexity reduction could potentially evolve over the lifespan, providing a balance of metabolic cost [48] to allow for an elaboration of model components in multi-modal regions. It could also contribute directly to the poor discriminability of (unimodal) sensory inputs observed in older adults [49], which in turn may preface as a ‘common cause’, age-related cognitive disruption [50].

From a physiological perspective, predictive coding may provide a useful process theory for neuronal computations in aging. For example, simulations of the mismatch negativity paradigm predict a rapid trial-by-trial suppression of evoked responses that rests on the neuromodulation of superficial pyramidal cells reporting prediction error. Previously, we confirmed this prediction empirically using dynamic causal modeling and a placebo-controlled study of cholinesterase inhibition [18]. In a complementary simulation study of frequency-based MMN, NMDA mediated synaptic plasticity has been shown to underpin model reorganization at the predictive cell population [17]. Given the therapeutic benefit of cholinesterase inhibition [51], and the role of NMDA receptors [52] in dementia further modeling of non-invasive psychopharmacological studies may provide important insights into the synaptic basis of age-related changes in perceptual processing.

## Methods

### Subjects

We studied 97 healthy volunteers, 55 female, who were cognitively normal with no neurological or psychiatric illness or serious medical history. Subjects were aged 20 to 83 and all completed the recording paradigm.

### Ethics Statement

Subjects were paid for their participation and consented to all procedures, which were conducted in accordance with the Declaration of Helsinki (1991). Protocols were approved by the South-East Strategic Health Authority Regional NHS Ethics Committee.

### Experimental Paradigm and MEG Data Acquisition

MEG recordings were made in a magnetically shielded room using a 275-channel CTF system with SQUID-based axial gradiometers (VSM MedTech Ltd., Couquitlam, BC, Canada). Recordings were obtained during two sessions with a small rest period between scanning, during which time subjects remained in the MEG scanner. Head localisation was performed at the beginning of each session.

Auditory responses were elicited by stimuli comprising pure tones presented binaurally over headphones. Two stimuli, at 500 Hz and 800 Hz were presented in a pseudo-random sequence for 70 msec with 10 msec rise and fall times. The first tone served as the standard and was presented on 88% of trials, while the second, which served as the oddball, was presented on 12% of trials. The sequence ensured that the minimal interval between oddballs was 2 trials and the maximum was 25 trials. The ISI was fixed at 1100 msec. Loudness was adapted to each subject's auditory threshold to be clearly audible binaurally – as measured in a test run while in the scanner. We collected data over two sessions for 96 subjects. For one subject we recorded just one session. Sessions were 6 minutes in length.

### Data Pre-processing and Source Localization

MEG data were first filtered off-line (band-passed from 0.5–30 Hz), down-sampled (to 200 Hz), epoched (from −150 ms to 350 ms peri-stimulus time), baseline corrected to 0 ms peristimulus time, artefact corrected (peak-to-peak threshold 5pF) and averaged to obtain event related fields (ERFs). The analysis routines we used are available in the academic freeware SPM8 (http://www.fil.ion.ucl.ac.uk/spm/).

For source localization, multiple sparse priors were used to estimate the cortical sources of the sensor recordings, using standard settings [53]. Multiple sparse priors employs several hundred patches of activation that are iteratively reduced until an optimal number and location of active patches are found using a greedy Bayesian search. A tessellated cortical mesh set in canonical Montreal Neurological Institute (MNI) anatomical space – as implemented in SPM8 – served as a brain model [54]. This dipole mesh was used to calculate the forward solution using a spherical head model. Source activity measures were then interpolated into MNI voxel space and analysed using statistical parametric mapping – at the between subject level – using an *F* test: A contrast of standard vs deviant stimuli was computed at *p<0.05* family-wise error corrected (Figure 2) based on the evoked power over frequencies from 0–30 Hz and from 60 to 300 msec peristimulus time.

### Dynamic Causal Modeling

For dynamic causal modeling, we used source location priors as described in previous DCM analyses of the mismatch negativity (MMN) paradigm [23], [25]. These included sources in Heschl's gyrus, superior temporal cortex and inferior frontal gyrus and were consistent with the source localisation analyses. The MNI coordinates were as follows: left HG: *x = −42, y = −22, z = 7*; right HG: *x = 46, y = −14, z = 8*; left STG: *x = −61, y = −32, z = 8*; right STG: *x = 59, y = −25, z = 8*; left IFG: *x = −46, y = 20, z = 8*; right IFG: *x = 46, y = 20, z = 8*. These prior locations were optimised at an individual level during DCM inversion using distributed dipoles and the forward solution from the above source localisation [55].

In DCM, event related fields are modelled as the response of a dynamic input–output system to exogenous (experimental) inputs [29]. The DCM generates a predicted ERF as the response of a network of coupled sources to sensory (thalamic) input – where each source corresponds to a neural mass model of three neuronal populations. Our dynamic causal models comprised hierarchical sources with prior locations as defined above, extrinsic input to primary sensory regions and extrinsic connections of forward and backward type [56]:

MEG sensor data were fitted over 0–300 msec peristimulus time, with the following model: auditory input (modelled as a Gaussian bump-function, with a prior onset of 64 msec) entered bilateral Heschl's gyrus, which provided forward connections to STG within each hemisphere. STG sent top-down backward connections to HG. STG also sent forward connections up to IFG and received backward type connections from IFG. To accommodate trial-dependent differences, stimulus specific parameters were included for all extrinsic connections. The neural mass model describing the activity of each source comprised three subpopulations, each assigned to three cortical layers – which determine how they receive external inputs [56]. Spiny stellate cells receive input via forward and thalamic inputs and are located in layer IV. Pyramidal cells and inhibitory interneurons are located outside of layer IV. These receive inputs from backward connections. Extrinsic output cells are the pyramidal cell subpopulation in each region.

The neuronal dynamics were based on a conductance based model with intrinsic AMPA receptors (at all cell populations), GABAa receptors (at pyramidal cell populations and inhibitory interneurons) and NMDA receptors (at pyramidal cell populations and inhibitory interneurons) [57] (specified as the “NMDA” model in the SPM interface). The DCM generates a predicted ERF as the response of the network of coupled sources to sensory input. This input takes the form of a narrow (16 msec) Gaussian impulse function, which accounts for some temporal smoothing in thalamic volleys.

For computational expediency, DCMs were computed following dimensionality reduction to eight channel mixtures or spatial modes. These were the eight principal modes of a singular value decomposition (SVD) of prior predictive covariance based upon the prior source locations. Note that data are normalized prior to model inversion and the forward model which accounts for source transmission to the MEG sensors is also parameterised and optimised during inversion.

### Analyses of Conditional Model Parameters

Where data were collected over multiple trial runs (96 out of 97 subjects), DCMs were fitted for each run separately and post-hoc conditional parameter means were computed using Bayesian parameter averaging (BPA). These were used for the regression models and lifespan correlation. BPA involves a weighted average where each model's posterior mean (in DCM.Ep) is weighted with its relative precision, where precisions are obtained from the inverse of the posterior covariance.
